# Perceptions and experiences of psychological trauma in nursing and psychiatric nursing students: A small scale qualitative case study

**DOI:** 10.1371/journal.pone.0277195

**Published:** 2022-11-03

**Authors:** Kathryn M. Chachula, Emma Varley

**Affiliations:** 1 Faculty of Health Studies - Department of Nursing, Brandon University, Brandon, Manitoba, Canada; 2 Faculty of Arts - Department of Anthropology, Brandon University, Brandon, Manitoba, Canada; Waikato Institute of Technology, NEW ZEALAND

## Abstract

Entry-level health care professionals are socialized to accept the norms and values associated with institutions in which violence and suffering is considered an anticipated and even routine and normalized part of frontline care. The objective of the study was to illuminate the subjective experience of psychological trauma in graduates from a baccalaureate nursing and psychiatric nursing program using the McGill Illness Narrative Interview, an ethnographic interview guide. Participants included graduates from each program in a western Canadian province who reflected back on their experiences of trauma as students and newly-graduated nurses within their first year of practice as a regulated health professional. Results: Six key themes were identified. Witnessing sudden change in patient or client status and unexpected death; Emotional labour; Faculty incivility; Sabotage, bullying and verbal abuse from the health care team; Exposure to physical violence and sexual inappropriateness; and Mobilizing supports. All exposures were linked to the participants’ definition of psychological trauma. Conclusions: The study findings highlight the power dynamic, abuses, and vulnerability between students, faculty, and their clinical counterparts without adequate recourse. There is a need to foster emotional intelligence, self-efficacy, and resilience when potentially traumatic and stressful experiences occur with student nurse and early-career nursing populations.

## Introduction

Students in undergraduate nursing and psychiatric nursing programs are future professionals entering a heath care climate rife with violence and anxiety-provoking life-and-death experiences. According to the Canadian Federation of Nurses Unions [1], nurses experience three-times more violence than police and correctional service officers combined. Within the western Canadian province of Manitoba, 25% of nurses consistently experience post-traumatic stress disorder (PTSD) symptoms [2]. Encountering stressful situations and bullying from both colleagues and superiors compromise the future of the nursing profession with newly-graduated nurses leaving practice to pursue other careers [3].

The goal of the study was to and identify the needs and vulnerabilities regarding perceptions and experiences of trauma within the Bachelor of Nursing (BN) and Bachelor of Science in Psychiatric Nursing (BScPN) student populations within a western Canadian province. The McGill Illness Narrative Interview (MINI) methodology developed by Groleau et al. [4] derived from medical anthropology was utilized to better understand how, when, and under what circumstances graduates of BN and BScPN programs self-identify as being ‘traumatized’, and/or act on their experiences and feelings of clinical-related ‘trauma’. All participants in the study described events that were deemed personally traumatic. The events described included an underlying vicarious trauma by virtue of simply caring for a patient or witnessing traumatic events unfold within their practice environment.

## Background

PTSD is one of several stress-related disorders outlined in the *Diagnostic and Statistical Manual of Mental Disorders* (DSM-V) [5]. PTSD is precipitated by a direct or indirect exposure to a traumatic event. This exposure results in a constellation of symptoms with a variety of aggravating and protective factors. Scholars have argued that there is a need to a understand how culture, race, gender, workplaces, institutional and professional cultures all may contribute to the very fabric that leads to development of traumatic illness, disorders, treatment, and stigma [6–11].

Workplace settings such as emergency departments where frequent traumatic events occur put health care providers that include nurses, physicians, and emergency responders at risk for PTSD and stress responses [2, 9]. Research findings have linked the development of PTSD to direct physical violence, the perception of serious threat, and witnessing severe injury or death of patients [9] which can occur in a number of different health care settings. The view that PTSD formation is an occupational hazard for combat veterans [11], nurses, and professionals that provide emergency services [9] requires exploration to understand aggravating and protective factors.

Social support from direct supervisors and close colleagues protected nurses against the intention to leave the nursing profession [12]. Conversely, studies centred on nursing students who experienced bullying from staff nurses, clinical instructors, student peers, as well as patients and their families found that these individuals were significantly more likely to leave the nursing program [13] and quite possibly the profession. In a similar study, students who experienced bullying or harassment suffered negative feelings of anxiety, inadequacy, anger, embarrassment, humiliation, depression and fear [14] which align with PTSD symptomology. These findings highlight a perpetual cycle of students and nurses working in occupational settings where a workplace culture of being threatened with physical harm and verbal abuse fueled by oppression, social hierarchies, and power dynamics [13, 14]. There is a need to understand how graduates from BN and BScPN programs defined a psychological traumatic experience while undertaking their undergraduate nursing education, understand their vulnerabilities as students, and the circumstances surrounding the traumatic events to inform how educators can engage students that promote resilience, emotional intelligence development, and coping skills. This study undertakes a detailed qualitative exploration of seven nursing student participants of traumatic experiences in an occupational setting.

## Materials and methods

### Study design

The *McGill Illness Narrative Interview* (MINI) developed by Groleau et al. [4] was used to guide data collection to elicit participants’ meaning of trauma within the socio-cultural-historical context. The MINI can be applied to any health problem that includes trauma-related illness. The semi-structured interview schedule incorporated broad dimensions of the illness experience, psychological trauma, that allowed the participant to discuss *explanatory models* or perceived causal linkages between the illness and health experiences, *prototypes* that allowed for exploration of the illness through use of analogy, and *chain-complexes* where multiple life events were or were not associated with the illness experience. A strength of the MINI is that it “is not limited to symptoms, symptom cluster, syndromes, biomedical diagnoses or popular labels” [4, p. 677] thus reducing any potential stigmatization and pathologizing of symptoms. This permitted recognition and validation of participants’ experiences in educational and health care systems that have inadequately prepared graduates to practice in a profession at risk of mental harm and psychological injury, without a diagnosis or diagnostic label that authorizes their suffering. For example, in the nursing profession, the diagnostic label of having PTSD has served to obtain health coverage for the treatment of experiencing traumatic events. The MINI is not a clinical interview, but an ethnographic interview in which the interviewee is the expert, not the clinician or researcher which served to empower the participant narrative. Accordingly, in-depth, semi-structured, individual interviews lasting one to three hours were conducted by the study authors and audio-taped by the researchers in 2018. Participants were provided an opportunity to review and validate their interview data, withdraw any data, and discuss preliminary results prior to the conclusion of the study. The data collected from participants focussed on their experiences as students enrolled in the BN or BScPN programs at one western Canadian university.

### Ethical considerations and recruitment

The study is in compliance with the *Canadian Tri-Council Policy Statement*: *Ethical Conduct for Research Involving Humans* [15]. Following receipt of ethical approval granted from the Brandon University Research Ethics Committee (Certificate: 22097), participants were recruited into the study. Since one of the study authors had prior contact with the students as a professor in the nursing program, the alumni association, a third-party, distributed a letter of invitation through an email list serve to limit any sense of coercion and researcher bias in the recruitment process. It is estimated that approximately 60 graduates with an active email address with the third party received the invitation to participate. Interested participants indicated their intent to participate along with their contact information, by return email to the corresponding author. Written informed consent for participation, audio-taping of interviews, and publication of study findings was obtained. Participants were provided the opportunity to review their interview transcripts to confirm and withdraw any data collected. No participants withdrew from the study once enrolled. Interviews occurred in a mutually agreed location in a quiet meeting room on the university campus. Ethical approval was granted to interview participants for one to three hours, up to three times. Narrative findings are presented without use of participant names or pseudonyms to protect confidentiality of participation. Study participants and members of the research team were encouraged to access psychological supports as needed throughout the study, through to the publication process and during any dissemination of the study findings. A third-party counselor trained in post-traumatic stress responses was recruited to offer study participants access to counseling support should the individual require immediate coping supports. Throughout the study, no participants accessed the counseling service.

### Sample and participants

Convenience and purposive sampling through the alumni association resulted in a final sample comprised of seven graduates from the nursing and psychiatric nursing programs within one year of program completion. Due to third-party recruitment, it is not known how many possible graduates were eligible to participate in effort to calculate a response rate. There were six females and one male participant. All participants self-declared as being gender-normative, identifying as either male or female with none identifying as non-binary. Participants reflected back on their experiences as students within their four-year baccalaureate program of study and within their first year of professional practice. The MINI elicited participants’ experiences and meaning of trauma using both open and semi-structured interview questions. All participants had a successful pass on their nursing licensing exam, secured employment as practicing nursing professionals, and had access to counseling services through their employer and study protocols if needed. Their ages ranged from 24–51 years (Mean age 30, SD 8.9 years), none reported as being Indigenous or from a visible minority. Irrespective of participant age, age was not a factor in being exposed to traumatic experiences.

### Data analysis

The analyzed data were derived from the verbatim audiotaped interview transcripts, confirmed by each participant, and field notes taken during the interview. Data collected using the MINI lends toward a variety of mixed-method and qualitative analysis approaches including grounded theory and thematic analysis [4]. For the purposes of the research study, Braun and Clarke’s [16] six-step approach to thematic analysis was used to analyze the data collected. The *Professional Quality of Life* theoretical model [17] informed the data analysis approach. Each investigator performed an in-depth, independent review of the data to identify themes, corroborate findings, and discuss common themes among the participants. Given the exploratory nature of the study, only themes that were saturated with data adequacy [18] are presented. Trustworthiness was an important aspect of data analysis, therefore, member checks with participants, newly-graduated nurses, and students were conducted to confirm the key study findings. The findings presented are adequate representations of participants’ own respective experiences. The investigators provided use of verbatim participant quotes to illustrate the major themes and thick description of the narratives collected. Many themes surrounding psychological trauma contained a grotesque nature to the experience suffered.

## Results

Following repetition of participant experiences, the research investigators defined a traumatic experience from the perspective of recent program graduates and six key themes were identified. They included: Witnessing sudden change in patient or client status and unexpected death; Emotional labour; Faculty incivility; Sabotage, bullying and verbal abuse from the health care team; Exposure to physical violence and sexual inappropriateness; and Mobilizing supports. All exposures were linked to the participant’s definition of psychological trauma. Interestingly, the participants invoked language that closely paralleled biomedical language in effort to describe their experiences, whether knowingly or not, that mirrors diagnostic criteria for PTSD and other mental health disorders. This may be reflective of their enculturation within the health disciplines.

Study participants defined a traumatic experience as “anything that causes emotional, physical, or mental distress.” The participants’ definition encompassed a broadly defined concept of trauma. One participant stated, “it’s the negative emotional feelings…like grief, anger, sadness, and guilt.” Others stated the negative feelings are comprised of “anxiety, fear, just heavy emotional stress” that “impacts you emotionally and kind of stays with you.” In addition, another participant stated, “it was a lot of emotional labour, I was so exhausted.” Lastly, participants described a traumatic experience as “its yours, and you experience it differently from other people.”

### Witnessing sudden change

Participants reported witnessing sudden change in circumstances surrounding death, decline in a patient or client, and a sudden change in status. In particular, involvement in a ‘Code Blue’ when a patient enters cardiac and/or respiratory arrest while in clinical, was a significant source of traumatic stress. This was particularly relevant for the participants given their vulnerability as a student when the events unfolded in the clinical practice environment. Participants reported being in a Code Blue as “sheer panic and chaos.” One participant reported, “All of a sudden, she went limp and her eyes rolled back and she voided everywhere.” Another participant stated:

It was like, a nightmare. Like it was, like, I just remember being, like, Oh, my God! Like, the whole time …. And I just felt so numb … just like, I have no idea what I’m doing here! Like I’ve never seen anything like that…. it’s traumatic, that was very traumatic, it was very traumatic.

Other participants reported how dealing with death was emotionally challenging in a variety of settings. As one participant reported, “I’d like never really experienced a person dying with me…. But I was just like, overwhelmed with emotion.” One graduate who was placed in a maternal-child setting during the fourth-year final senior practicum stated, “The deaths, the people with really hard pregnancies that lost their babies, like, it was hard to see…”. Preparing the body was also difficult as per one participant reflected, “we got the body somewhat as ready as we could…and it was just the strangest thing where you wrap them in plastic.” Another reflected, “when you don’t deal with dying people on a regular basis it is traumatic”.

When a patient experienced an unexpected, sudden change in status the participants all reflected feeling scared, guilt, and not sure of what to do. One stated, “… It was really scary. The first time it happened I panicked.” Another reflected:

It was really stressful, uh, there was just, I’ve never seen so much blood in my life before…. Um, I found out, you know, a few days later that he [the patient] didn’t make it, I went, like, to the back room and, like, sat and had a coffee and I like, just cried. Because I was like, ‘What just happened?’…to have somebody go from, like, conversing with you to unconscious and intubated and, you know, within a few hours, was just, like … emotionally frazzled and not really sure how to process things.”

Students do not routinely encounter death and rapid changes in patient status where they are expected to intervene as a member of the health care team. Many relayed feeling alone and unsupported through university structures to assist in processing appropriate and inappropriate actions, as well as understanding when any interventions taken, may be futile.

### Emotional labour

All participants described regulating their own emotions while witnessing suffering as a student in their program. Many described trying to portray a professional appearance and struggling with not being sure of what to say or engage with family members of the patient. As per the previous theme that highlights death and dying, some participants questioned, do you cry with the family when your patient dies? The majority of participants reflected on keeping emotions separate from patients and family members to maintain emotional distance as they wrestled with professional boundaries.

Other participants continued to wrestle with past events, not knowing if they said or did the right thing in life-and-death, highly charged situations. In particular, one participant reflected back on an event that occurred in their second year of the program, “the guilt has lessened over the years and over the things that I’ve done and learned. But it’s just, maybe I could have said something different; you know?” Another stated, “I was a new student and I was wondering if there was something maybe I should have done differently.” One participant reflected on their experience after a patient had an unexpected decline stating, “The family had a really hard time coping, and that was really hard. I didn’t know how to respond to the family so that was really hard for me too, seeing them go through that.” After a Code Blue, another participant stated:

You like go back to these rooms and [the patients] are like ‘my call bell’s been going off for 30 minutes what have you been doing’, and you can’t exactly go like, ‘Oh somebody died.’ Like, sorry I didn’t answer your bell for your ginger ale. So, you have to be like: ‘Other patients needed me at the time, I will get to you what do you need now, I am here.’

These findings highlight an ongoing compartmentalizing of actions and behaviours under the guise of maintaining a professional identity and acting with compassion. Yet, at the same time, continuing to ruminate and reflect back on past events, containing, and managing unsettled emotions during and long after the event took place.

### Faculty incivility

Several of the participants reported experiencing incivility and bullying behaviours from faculty members and instructors that led to psychological trauma in classroom environments. For example, one reflected:

I had a professor ostracize me in the middle of class… I got an email telling me to come to their office… When I sat in the office they went, ‘I literally can’t stand you.’ Which I’m pretty certain is not an appropriate thing to be telling your students.

As a result of faculty incivility, participants reported withdrawing from participating in class. “You don’t want to be a part of it, and you just kind of put your head down and keep going.” When participants were asked how they managed faculty incivility, many felt unprepared and unwilling to pursue any complaints. As described by one participant:

I would never confront a professor. I just wouldn’t. I’ve said before though, I really dislike conflict. But I just wouldn’t. They have so much influence over how you are doing in your schooling that I just wouldn’t. [Talking to] a neutral party would be probably beneficial.

Other participants described simply putting up with the incivility with one who stated, “I’m just going to grit my teeth and bear it basically”, ostensibly for fear of the impacts on their continued progress in the nursing program. The self-silencing expected of students by higher education left participants disempowered and vulnerable to continued abuse when wrongdoing occurs. These findings highlight the power dynamic and vulnerability between students and their instructors without adequate recourse. Students not only suffer incivility in the university setting, but also reported abuse in the clinical practice environment.

### Sabotage, bullying, and verbal abuse

Participants in the study experienced sabotage, bullying, verbal abuse, and being the subject of rumours while undertaking their studies from members of the health care team. Participants reflected on experiences with nurses, physicians, as well as patients. With regard to nurses, one participant reflected “the nurse started this whole rumor that a student had pushed a patient… [as a result, the nurses] they pretty much stripped us down to being able to do *nothing* [sic]. Um, and just like lying about us.” Other participants reported an unfriendly clinical environment stating “we never knew if they were going to fly off the handle or just whatever, they didn’t want to interact with us”, “there was a kind of cattiness, emotional things, and nurses tend to eat their young.” One participant elaborated stating “I felt like she [the nurse] just didn’t see me as, umm, I don’t know, somebody who was even there.”

Physician behaviours in the clinical setting were also described as abusive by study participants. One participant described experiencing being yelled at by a physician as a regular occurrence in the practice setting. “Doctors yell at you, and sometimes that’s sometimes more traumatic than anything else, just being yelled at by somebody.” Another participant reported:

[The doctor] He got this close to me [holds hand in front of face], and yelled at me in front of this family… At that moment I checked out, I wasn’t even in the room anymore. All I could think of was, I want to leave…like, what point did that make? Was he trying to show he’s smarter than me…?

During a Code Blue experience, one participant as a second-year nursing student, in their first medical placement of the program reported emotions running high during the following circumstance:

I just remember, the ICU [intensive care unit] doctor standing there screaming ‘Where’s the nurse, who knows this patient?’ And I remember having my little concept care map [nursing care plan] and standing there… and I remember standing there, like, shaking.

These examples highlight a physician culture of continued hierarchal violence between nurses and physicians, with nursing students also being the subject of and bearing witness to a health care culture of incivility. Shapiro [27] argued underlying this abuse is an engendered nature of events and “physician arrogance” (p. 4) where in all the situations described by the participants, a male-paternalistic, dominating physician presence, that exists within the health care system was reported to the investigators.

Unfortunately, the study participants also reported experiencing verbal abuse from patients. As one reported, “She was calling me constant names, some really, really, the worst things you could imagine, just constantly for an hour… I left the room and I was actually—like visibly shaking from the stress of being in the room.” Another stated, “The patient was, uh, really angry at me and screaming. And it was because they wanted some more Lorazepam [anti-anxiety medication] and when I asked them what was going on and they were, like, “Give me my Lorazepam!” [loud, yelling voice]. Among the study participants who described abuse from patients, all expressed that experiencing verbal insults were simply “part of the job.”

### Exposure to physical violence and sexual inappropriateness

Participants in the study mainly reported being exposed to physical violence and sexual inappropriateness during their final, fourth-year senior practicum experience. Two participants placed in a psychiatric care area described putting up with verbal and sexual advances from a patient stating, “I would go to change his diaper because he was incontinent. And he would be like, ‘Hey do you give hand jobs too?’ I’m like, ‘no [shakes head], no.’” Another described “… [the patient] took them [the medications] from me and then threw them and the water cup at me. And I was like, ‘Okay, I guess this is how we’re starting the morning.’”

During times when a ‘Code White’ was called for a physically abusive or aggressive patient, participants reported feeling highly unsettled. One participant reflected seeing staff administer a needle against a patient’s will, “the first time I saw that—that was pretty, like, um, it’s really intense the first time you see something like that happen, because it doesn’t feel humane at all…but it still affects you emotionally.” One described a different scenario when “… [a patient] lunged towards us… We ended up having to seclude him and giving him a needle, that kind of thing. But I was *so* [sic] afraid…. I don’t know, it was so scary.” Another reported, “So like, you call security and you sedate them… you’re like traumatized after you’ve been abused by your patients.”

Participants placed in an emergency department setting described caring for inebriated patients. One stated “I caught one smoking meth in the bathroom…” another described having a “grabby patient”. The participant further reflected, “I know its inappropriate, but nothing’s going to come of it. They are drunk and grabby…One guy grabbed my butt, and working I’ve had guys grab my boob…it’s one of those things.” Physicalness in the clinical setting was also described by another participant when trying to describe her day for her partner, “I’d tell my fiancé, yeah a guy today just tried to grab my ass, and an old man tried to punch me today …. it’s part of my job. It shouldn’t be, but it is.” When asked how the partner responded, the participant stated, “My fiancé was like, ‘are you okay?’ I’m like, ‘yup.’” [stated with sarcasm]. Again, participants described a health care culture where experiencing different forms of violence was normalized as part of their work as entry-level professionals.

### Mobilizing supports

Participants described mobilizing their interpersonal and counselling supports following involvement in traumatic incidents. The majority of RN participants expressed that they learned to cope with the nursing role after completing their program, highlighting a gap in the BN program. Those who graduated from the BScPN program indicated they learned some coping mechanisms during their education. Several participants reported talking with a family member or relative who works in the health care field, but hesitated speaking with someone not familiar with the health care arena. One participant stated, “I’m thankful that my mom’s a nurse. After every shift, I would call her when I’m driving home…. I called my mom crying going, ‘What have I done, I hate this floor, I hate my job, I’ve made a terrible mistake.’” Another participant described talking with their aunt “… she just kind of heard me out and kind of validated some feelings I had without really actually saying a whole lot.” Participants reported feeling hesitant to talk with non-health care friends and family. “[My partner] has no idea, like, he doesn’t understand. He’ll try and do his best but, um, I didn’t want to put that on him because I chose to go into this profession.”

To cope with the role of the nurse, participants reported different positive personal health behaviours ranging from physical exercise, visiting friends and family, mindfulness techniques, and use of medication. As one participant reflected:

I do things to cope in day-to-day life. I go to the gym; I find that rewarding for myself. I’m very close with my family, I’m able to speak to them. I have a good friend group. I just live my everyday life.

When asked to described what mindfulness techniques are useful for participants, one reported “deep breathing and, uh, I should do more but it’s hard, I haven’t done a body scan in a while, I do like coloring, um, I actually have a coloring book at my desk which is awesome.” One example of medication use described by a participant involved use of a sleep aid. They stated, “I don’t need sleep aids on my days off but when I’m working, it’s like I actually need a sleep aid to make sure that I get my sleep so that I can get through the day.” These findings highlight and confirm that many students and nurses are left to their own devices, to cope with traumatic experiences.

## Discussion

When considered altogether, participants’ accounts confirm the varied ways that suffering and trauma arose simultaneously from and, in turn, impinged on their nursing education and health care provision. In describing their experiences of incivility, abuse, and critical incidents such as Code Blue and Code White events, participants confirmed they were not only poorly protected from interpersonal and emotional stressors, and the risks of traumatic exposure and re-exposure. Many also described themselves as being actively discouraged from drawing attention to their own needs for emotional and physical safety and security. In relaying how often they were “left alone” to face the consequences of upsetting, disturbing, and traumatizing events, participants described how the risks, harms, and emotional struggles they faced in the course of their studies and nursing role were implicitly cast by supervising faculty and clinicians as being mundane and quotidian, or insignificant in nature. Even more, participants made it a point to emphasize the lack of positive support and effective intervention they received from faculty and health care personnel after critical incidents, which further suggests general inattention to and disregard of student and early-career nurses’ needs. It also signals the insufficiency or absence of system-side reforms, protections, and mental health support available to student nurses, and expectations that they prioritize professional boundaries and values ahead of their own experiences of suffering.

Harassment, abuse, assault, and violence are, to a worrying degree, not simply normalized but tolerated in educational and clinical practice settings. According to our participants, nursing education underprepares students for the wide array of critical incidents that they are likely to encounter during their practical and in their post-graduate work as early-career nurses. Early-career nurses are similarly socialized [19–22] to accept critical incidents and their emotional aftermaths, and their undermanagement and under-recognition by employers, as ‘routine’ and ‘normal’ elements of frontline care. Rather than acknowledging the distress resulting from maltreatment or shocking and tragic events, institutions encouraged students to rationalize critical incidents as unavoidable and, therefore, somewhat beyond the scope of institutions’ responsibility. For example, acts of violence were explained as the being solely the result of patients’ underlying mental and medical illnesses, rather than a failure to protect nurses from harm. We approach the deliberate neglect or disavowal of nurses’ needs following such events as examples of another kind of violence; namely, the horizontal [23, 24] and lateral violence [25–27]. Horizontal violence includes hostile and uncivil behaviours perpetuated laterally across nursing groups or within a hierarchy by educators and employers against student or early-career nurses, sometimes referred to as vertical or hierarchical violence [28]. The impact of horizontal and lateral violence experienced by early-career nurses can lead to physical manifestations of illness, increased sick days, diminished confidence or self-esteem, psychological impacts such as humiliation and anxiety, disillusionment with the nursing profession, as well as PTSD [29].

When articulating the seriousness of their experiences of distress and suffering, the participants relied heavily on biomedical and psychiatric languaging and diagnostic terms of reference. We hypothesize that, by medicalizing their varied emotional experiences and needs, the study participants made productive and meaningful use of the biomedical criteria in which they were educated and enculturated, and worked to accord greater credibility and gravity to their accounts. In relaying their experiences using medical language and terminology, we surmise this allowed participants the freedom to disclose their experiences, albeit within the confines of enculturated, disciplinary language. Notably absent from many participants’ accounts were discussion of the broader socio-structural and system-side politics and organizational cultures that placed them at regular risk of exposure to traumatizing critical incidents. Participants indexed the seriousness of their situation by emphasizing the magnitude of its psychosocial, physical, and psychiatric impacts. However, ‘distress’ and ‘suffering’ alone were not always sufficient to achieve necessary recognition and support. Diagnoses associated with higher degree of morbidity, such as PTSD, can be career-limiting or -ending, and are more likely to prompt institutions, including those otherwise-disinclined to intervene on lower orders of suffering, to provide necessary redress and resolution [30, 31]. By only responding primarily or exclusively to critical occurrences, institutions risk neglecting providers’ larger experiences of suffering and the events that give them rise, which, overtime and with additional exposures, can yield significant forms of cumulative distress, burnout, and PTSD.

Our participants’ accounts require that we assess the dynamically affective ways that universities and clinical practice environments can serve as sites of inequity, injustice, and violence, in addition to sources of persisting, abiding, and too-often unresolved trauma. In so doing, we gain valuable opportunities for acknowledgement, intervention, healing, and evidence-based insights into the ways nurses and students can be better protected from the critical incidents which, overtime, yield life- and career-altering traumas [32–34]. These can then be applied to develop nurse and student-centred strategies that ensure their safety and continued professional development, praxis, and better support to not merely survive, but thrive within the nursing profession [35, 36].

### Implications for practice

There is a need for nurse educators and policy makers to develop strategies that foster resilience, self-efficacy, and healthy coping mechanisms in undergraduate nursing students prior to their entry into the workforce [37]. Fostering emotional intelligence, self-efficacy, and resilience requires the development of policy and guidelines that attends to debriefing procedures for faculty and clinical instructors when potentially traumatic experiences occur with student nurse populations. Experiences of trauma have been compounded due to added stressors imposed by the novel coronavirus pandemic (SARS-CoV-2), otherwise known as COVID-19 [38]. Nursing students have experienced declining mental wellness and resilience due to factors that included feeling added anxieties related to clinical practice within a pandemic [39]. These findings highlight the need for nursing education institutions to embed policy and trauma-informed teaching practices to prepare and assist students engaged in the practice environment.

The investigators recommend normalizing the experience of trauma and psychological responses in effort to encourage peer support and positive coping mechanisms. The following guideline available in Table 1 was developed based on documents published by agencies that included the Academy of Medical Royal Colleges [38], Australian Centre for Posttraumatic Mental Health [40], British Psychological Society [41], United Kingdom Psychological Trauma Society [42], United States Department of Veteran Affairs [43], and the World Health Organization [44]. Ongoing dialogue between health care and education institutions is required that enable safe learning environments free from verbal abuse, violence, and re-course if potentially traumatic events occur in clinical practice settings affiliated with university institutions. The guideline can be implemented across international practice contexts that acknowledge stressors experienced during the pandemic, distressing physical or mental health experiences, and potentially traumatic events.

**Table 1:** Psychological well-being guideline for pre-professional health care students.

**Key Approaches**
**1. Normalize having a psychological response**. It is important for those exposed to a psychologically traumatic event that having a stress reaction is a normal response in an otherwise abnormal situation. This should not imply that the student health care worker is not a ‘good fit’ for the nursing profession or ‘weak’ given an unprecedented occurrence, or encountering a new learning situation that the learner has not previously experienced.
**2. Encourage supportive peer relationships that foster well-being**. This includes developing a supportive teaching-and-learning atmosphere among student peer-to-peer relationships, as well as students with members of the health care team, and clinical faculty. Post-secondary learning institutions should provide learners with the opportunity to resolve faculty incivility that does not jeopardize student progress within their field-of-study. Provide a supportive atmosphere that includes allowing students to engage in open, honest dialogue with their instructors without the fear of failure. Allow students to voice concerns and, in turn, address questions and devise a plan to diminish fears and any feelings of uncertainty. This may include changing the student’s assignment to one of light duties if a traumatic event has occurred while in the clinical practice setting. Pairing a student with another can also diminish the sense of feeling alone, and fosters positive teamwork and engagement. Encourage students, faculty, and members of the health care team to speak openly about their experiences. This allows all parties to mentally process what has occurred, allows leaders to assess the well-being of those involved, and refer for support if necessary.
**3. Provide psychological first aid training and education for students, faculty, and members of the health care team**. Psychological first aid training offers learners the chance to normalize stress reactions, recognize when they are occurring, know how to encourage the individual to mobilize their own coping supports, and know when to refer if coping mechanisms are ineffective or disrupted. Importantly, if the individual is adequately coping, offering psychological intervention too soon (such as one-off critical incident stress debriefing) can disrupt positive coping strategies and interfere with the individual’s mobilization of personal resources.
**4. Promote access to well-being services available to students, faculty, and members of the health care team**. Students in health-related disciplines may face compounding stressors related to university studies, alternating classroom-laboratory-clinical schedules, and day-to-day stressors. Ensuring that access to resources that promote mental health and well-being are essential to facilitate access to supports when referral to services is warranted and positive coping mechanisms are in jeopardy. Encourage use of positive coping practices used in the past to navigate the current situation. Acknowledge that a stress response can be dehydrating and encourage re-hydration with water. Avoid substance use or risky behaviours that can interfere with positive coping. Engaging in light exercise following a traumatic event can assist in self-care and coping following the launch of a flight-or-fight stress response. Encouraging healthy eating, exercise, sleep/rest patterns, mindfulness, and time management skills can play an integral role to psychological safety. Again, relay that feeling stressed is a normal reaction given the situation. Seek social support as needed, including through digital means when physical interaction is limited within the COVID-19 climate.
**5. Prepare for stress and potentially traumatic events**. Educators ought to prepare their students for stressful encounters and potentially traumatic experiences in the health care system. While some forms of stress may be anticipated, others may occur without warning and/or adequate preparation. Therefore, communication skills related to de-escalation techniques, mindfulness and self-monitoring for stress reactions, coping skills, addressing incivility in the workplace, as well as caring for a patient related to death and dying should be integrated into preparational curricula prior to entry into the workforce. Integration of ethical decision-making within curricula is paramount in a climate where a lack of resources and personal protective equipment can lead to moral distress. Encourage students, faculty, and members of the health care team to check-in with colleagues, focus on what you have control over to lower anxiety, and foster positivity and hope in your practice that promotes resilience.
**6. Adopt trauma-informed approaches to care and education**. While it is beyond this guideline to provide an exhaustive list of traumatized populations, it is important to recognize the need for trauma-informed approaches when delivering health care and in education practices. In Canada and internationally, members of the 2SLGBTQI community, Indigenous peoples, and persons of colour have faced institutional bias and racism in a variety of sectors that contribute to psychological trauma. In addition, emergency services personnel, military and ex-military personnel are known to have high exposures of trauma. As health care workers and educators, it is important to acknowledge your own and others’ prior trauma history that may include childhood sexual or physical abuse, domestic violence, government or societal persecution, and/or a combination of prior traumatizing events that may have a cumulative and/or ongoing effect. Establishing trust and rapport is essential. In addition, upholding and explaining the covenants of care relating to confidentiality, privacy, and consent are paramount to establishing trust and rapport in adopting a trauma-informed approach that recognizes prior exposures of trauma.

## Limitations

This pilot study serves as an important initial investigation that sought to understand perceptions and experiences of trauma within an undergraduate nursing student population. The small sample size of seven participants introduces selection bias where only those with self-described psychological trauma may have agreed to participate in the study, thus limiting representativeness of the population and generalizability of research findings. However, given that there is a lack of published studies that explore psychological trauma in pre-licensure health studies students, the findings offer the research and health care practice community tangible evidence that traumatic stress is occurring within pre-professional student groups. Pye et al. [45] argued that “If the study is novel, it may add to the literature regardless of sample size” (p. 6). Data adequacy was met through use of thick description relevant to the phenomena of interest, in addition to member checking, triangulation with published studies, reflexivity, and researcher peer-debriefing [18]. The participant characteristics limit the findings to those who present as Caucasian and from a moderate socio-economic standing. Specific narratives related to participant faith and/or spirituality were not collected and did not arise within the interviews. The results do not capture black, indigenous, people of colour (BIPOC) experiences from those who identify as a visible minority or who openly identify as being part of the 2SLGBTQIA+ community. Future research should include quantitative studies that assess the prevalence of stress, psychological trauma, and incivility with robust analysis that includes demographic factors such as age, ethnicity or race, gender, and role within the health care team.

## Conclusion

This study affirms that nursing student populations are exposed to traumatic events in the health care setting while undertaking studies in preparational programs. The neglect and disavowal of student nurses’ experiences and needs confirm how institutions permit structural violence and disadvantage that impact the success of nurses’ education and professionalization. Due to the nature of student placements in health care environments during the course of baccalaureate curricula, it is imperative that employers consider the experience of students while enacting professional roles and responsibilities while engaged in nursing work within clinical practice environments.

## Supporting information

S1 Checklist (DOC)

S1 File (PDF)
