# Supplementary material for: Perceptions and experiences of psychological trauma in nursing and psychiatric nursing students: A small scale qualitative case study
Source: PLoS One. 2022 Nov 3;17(11):e0277195. doi: 10.1371/journal.pone.0277195 (PMC9632886; doi:10.1371/journal.pone.0277195)
Supplement: S1 Checklist — (DOC) [file pone.0277195.s001.doc]

**Manuscript:** Perceptions and experiences of psychological trauma in nursing and psychiatric nursing students: A small scale qualitative case study

PONE-D-21-16746R1

**Consolidated criteria for reporting qualitative studies (COREQ): 32-item checklist**

Developed from:

Tong A, Sainsbury P, Craig J. Consolidated criteria for reporting qualitative research (COREQ): a 32-item checklist for interviews and focus groups. *International Journal for Quality in Health Care*. 2007. Volume 19, Number 6: pp. 349 – 357

| **No. Item** | **Guide questions/description** | **Section(s)/ Page # Reported** |
| --- | --- | --- |
| **Domain 1: Research Team and Reﬂexivity** | | |
| *Personal Characteristics* |  |  |
| 1. Inter viewer/facilitator | **Which author/s conducted the inter view or focus group?**  Both authors participated interviews | Study design section  (p. 6) |
| 2. Credentials | **What were the researcher’s credentials?**  Both researchers have graduate degrees that include qualitative methodologies. The lead author completed their Master’s degree with a focus on qualitative grounded theory interviewing. Both have PhDs, one in Nursing, the other in Medical Anthropology with expertise working with traumatized and vulnerable populations. | Inferred in the Title Page  ORCID ID for the lead author is provided in Letter to the Editor to view scholarly profile and publication history. |
| 3. Occupation | **What was their occupation at the time of the study?** Professors at the university where the study took place. | Title Page &  Ethical considerations and recruitment section (p.6) |
| 4. Gender | **Was the researcher male or female?**  Both researchers are female. | Title Page |
| 5. Experience and training | **What experience or training did the researcher have?** Both researchers have experience conducting qualitative interviews. Both study authors have a number of papers published that include qualitative and quantitative research methods. | Not disclosed in paper |
| *Relationship with participants* |  |  |
| 6. Relationship established | **Was a relationship established prior to study commencement?** One researcher works in the Department of Nursing, hence a third party was used to recruit potential participants. | Ethical considerations and recruitment section (p. 6) |
| 7. Participant knowledge of the interviewer | **What did the participants know about the researcher?** 57% or 4/7 participants knew one of the study authors as a professor in the nursing program. | Inferred in the Ethical considerations and recruitment section  (p. 6) |
| 8. Interviewer characteristics | **What characteristics were reported about the interviewer/facilitator? (e.g. Bias, assumptions, reasons and interests in the research topic).** The background section of paper illustrates assumptions, reasons, and interests in the research topic. Pejorative statements raised by Reviewer 1 were removed from the Background section. | Background section  (p. 4) |
| **Domain 2: Study Design** | | |
| *Theoretical framework* |  |  |
| 9. Methodological orientation and Theory | **What methodological orientation was stated to underpin the study? (e.g. grounded theory, discourse analysis, ethnography, phenomenology, content analysis).** Ethnography - The *McGill Illness Narrative Interview* (MINI) with thematic analysis. The *Professional Quality of Life* theoretical model was used. | Study design (pp 5-6) & Data analysis (p. 8) |
| *Participant selection* |  |  |
| 10. Sampling | **How were participants selected? (e.g. purposive, convenience, consecutive, snowball).** Convenience sampling through the alumni association. | Sample and participants  (p. 7) |
| 11. Method of approach | **How were participants approached? (e.g. face-to-face, telephone, mail, email).** Approximately 60 were approached via an email listserve maintained by the university alumni association (a third party). | Ethical considerations and recruitment  (p. 6) |
| 12. Sample size | **How many participants were in the study?**  Seven. | Sample and participants  (p.7) |
| 13. Non-participation | **How many people refused to participate or dropped out? Reasons?** No participants withdrew from the study once enrolled. | Ethical considerations and recruitment  (p.6) |
| *Setting* |  |  |
| 14. Setting of data collection | **Where was the data collected? (e.g. home, clinic, workplace).** Mutually agreed location: University campus. | Ethical considerations and recruitment (p. 6) |
| 15. Presence of non-participants | **Was anyone else present besides the participants and researchers?**  Only the researchers met with participants | Inferred within the Study design section  (p. 6) |
| 16. Description of sample | **What are the important characteristics of the sample? (e.g. demographic data, date).** Included in the sample and participants section. | Sample and participants  (p. 7) |
| *Data collection* |  |  |
| 17. Interview guide | **Were questions, prompts, guides provided by the authors? Was it pilot tested?**  Semi-structured guide was used as outlined in the *McGill Illness Narrative Interview* (MINI) developed by Groleau et al. Reference to the MINI is provided in the reference section. The publication includes the interview guide. | Study design (p. 5) &  Reference sections  (p. 25) |
| 18. Repeat interviews | **Were repeat interviews carried out? If yes, how many?** Ethical approval was granted to interview participants up to three times - this included member checks. | Ethical considerations and recruitment  (pp. 6-7) |
| 19. Audio/visual recording | **Did the research use audio or visual recording to collect the data?** Audio recordings. | Data analysis (p. 8) |
| 20. Field notes | **Were ﬁeld notes made during and/or after the interview or focus group?** Yes, as stated in data analysis section. | Data analysis (p. 8) |
| 21. Duration | **What was the duration of the interviews or focus group?** 1-3 hours. | Ethical considerations and recruitment  (pp.6-7) |
| 22. Data saturation | **Was data saturation discussed?** Yes. Given the exploratory nature of the study, only themes that reached data saturation with data adequacy were reported in the results | Data analysis (p.8) |
| 23. Transcripts returned | **Were transcripts returned to participants for comment and/or correction?** Yes | Ethical considerations and recruitment (p. 6) & Data analysis (p. 8) |
| **Domain 3: Analysis and Findings** | | |
| *Data analysis* |  |  |
| 24. Number of data coders | **How many data coders coded the data?**  Both researchers (two). | Data analysis (p. 8) |
| 25. Description of the coding tree | **Did authors provide a description of the coding tree?** The themes with accompanying narratives are provided in the supplemental Supporting Information Data | See Supporting Information Data for PLOS ONE file. |
| 26. Derivation of themes | **Were themes identiﬁed in advance or derived from the data?** Themes were derived from the data. | Data analysis (p. 8) |
| 27. Software | **What software, if applicable, was used to manage the data?** No software was used. | Not applicable. |
| 28. Participant checking | **Did participants provide feedback on the ﬁndings?** Yes, member checks were completed. | Data analysis (p. 8) |
| *Reporting* |  |  |
| 29. Quotations presented | **Were participant quotations presented to illustrate the themes/ﬁndings? Was each quotation identiﬁed?**  Lengthy participant quotes are provided in the manuscript. | Results section  (pp. 9-17) |
| 30. Data and ﬁndings consistent | **Was there consistency between the data presented and the ﬁndings?** Yes, refer to results section and supplemental file that contains participant narratives. | Results section  (pp. 9-17) &  Supporting Information Data for PLOS ONE file. |
| 31. Clarity of major themes | **Were major themes clearly presented in the ﬁndings?** Yes, they are reported in the results. | Results section  (pp. 9-17) |
| 32. Clarity of minor themes | **Is there a description of diverse cases or discussion of minor themes?** Given the small sample size, only saturated themes with data adequacy are presented. Limitations regarding generalizability are included in Limitations section. | Results section  (pp. 9-17) &  Discussion  (pp. 17-20) &  Limitations (p. 23) |
