# Supplementary material for: Perceptions and experiences of psychological trauma in nursing and psychiatric nursing students: A small scale qualitative case study
Source: PLoS One. 2022 Nov 3;17(11):e0277195. doi: 10.1371/journal.pone.0277195 (PMC9632886; doi:10.1371/journal.pone.0277195)
Supplement: S1 File — (PDF) [file pone.0277195.s002.pdf]

# Perceptions and Experiences of Psychological Trauma in Nursing and Psychiatric Nursing Students: A Pilot Qualitative Study

Kathryn Chachula RN, BN, MN, PhD
chachulak@brandonu.ca
Canadian Nurses Foundation Scholar

Supporting Information Prepared for:
*PLOS ONE* – Data Availability

BRANDON UNIVERSITY

# Psychological Safety

*This content contains potentially triggering material.*

*To ensure a safe space, please take measures to promote your own psychological safety in review of the de-identified data.*

# Acknowledgements

Co-Author: Dr. Emma Varley - Brandon University

This project is supported by a grant from the Research and Workplace Innovation Program of the Workers Compensation Board of Manitoba

WCB
Workers Compensation Board of Manitoba

# Content

- Research Objectives
- Methodology
- Supporting Information with de-identified data

BRANDON UNIVERSITY

# Research Objectives

- Understand participants' definition of 'trauma'
- Identify vulnerabilities of nursing and psychiatric nursing students
- Understand circumstances that contributed to 'trauma'

# Methodology

McGill Illness Narrative Interview (MINI) (Groleau et al., 2006):

– ***Not a clinical interview, rather an ethnographic interview***

- Medical Anthropology
- Interview schedule: elicit meanings, health behaviours, and modes of reasoning related to the experience of illness
- Constructivist philosophical underpinning
- Elicited narratives shaped by memory, emotional regulation, and internal censorship within socio-political-cultural contexts
- Permits a variety of data analysis approaches

BRANDON UNIVERSITY

# Supporting Information

Major themes:

- Witnessing sudden change in patient status & unexpected death
- Emotional labour
- Faculty incivility
- Sabotage, bullying & verbal abuse from the health care team
- Exposure to physical violence and sexual inappropriateness
- Mobilizing supports

**All exposures were linked to the participant's definition of trauma**

BRANDON UNIVERSITY

# Supporting Information

Defining a ‘Traumatic Experience’:

- “Anything that causes emotional, physical, or mental distress.”
- “It’s the negative emotional feelings…like grief, anger, sadness, guilt.”
- “…anxiety, fear, just heavy emotional stress.”
- “…impacts you emotionally and kind of stays with you.”
- “It was a lot of emotional labour so I was exhausted.”
- “…it’s yours, and you experience it differently from other people.”

# Witnessing Sudden Change

Unexpected death, decline, and change in status.

Being in a code blue [cardiopulmonary arrest]:

- “All of a sudden, she went limp and her eyes rolled back and she voided everywhere.”
- “…it was just sheer panic and chaos.”
- “…it was like, a nightmare. Like it was, like, I just remember being, like, “Oh, my god!” Like, the whole time …. And I just felt so numb … just like, “I have no idea what I’m doing here!” Like I’ve never seen anything like that…. it’s traumatic, that was very traumatic, it was very traumatic.”

BRANDON UNIVERSITY

# Witnessing Sudden Change

Dealing with death:

- "…when you don't deal with dying people on a regular basis it is traumatic."
- "The deaths, the people with really hard pregnancies that maybe lost their babies, like, it was hard to see…"
- "I'd like never really experienced a person dying with me….But I was just like, overwhelmed with emotion."
- "We got the body somewhat as ready as we could…and it was just the strangest thing where you wrap them in plastic."

BRANDON UNIVERSITY

# Witnessing Sudden Change

## Dealing with decline and sudden change in patient status:

- “It was really scary. The first time it happened I panicked.”
- “It was really stressful, uh, there was just, I’ve never seen so much blood in my life before…. Um, I found out, you know, a few days later that he didn’t make it, I went, like, to the back room and, like, sat and had a coffee and I like, just cried. Because I was like, ‘What just happened?’”
- “…to have somebody go from, like, conversing with you to unconscious and intubated and, you know, within a few hours, was just, like … emotionally frazzled and not really sure how to process things.”

BRANDON UNIVERSITY

# Emotional Labour

## Regulating emotions while witnessing suffering:

- “You like go back to these rooms and these people are like ‘my call bell’s been going off for 30 minutes what have you been doing’, and you can’t exactly go like, ‘Oh somebody died.’ Like, sorry I didn’t answer your bell for your ginger ale.  So you have to be like “Other patients needed me at the time, I will get to you what do you need now, I am here”.
- “The guilt has lessened over the years and over the things that I’ve done and learned. But it’s just, maybe I could have said something different, you know?”

# Emotional Labour

## Regulating emotions while witnessing suffering:

- "The patient had kind of an unexpected decline. So the family had a really hard time coping, and that was really hard. I didn't know how to respond to the family so that was really hard for me too, seeing them go through that."
- "I was a new student and I was wondering if there was something maybe I should have done differently."
- "Somebody died like, unexpectedly. And that family is probably grieving, like, and their lives are changed forever, right? Like, that family member will never understand…"

# Faculty Incivility

## Transgressions from faculty members and instructors:

- “I had a professor ostracize me in the middle of class… I got an email telling me to come to their office… When I sat in the office they went, ‘I literally can’t stand you.’ Which I’m pretty certain is not an appropriate thing to be telling your students …”
- “I was unexpectedly called out to the front of the class to explain my situation…it was really unexpected and, you know I am fine, but maybe other people wouldn’t have been able to do that and would have been very upset by it.”
- “…[in class] you don’t want to be a part of it, and you just kind of put your head down and keep going.”

BRANDON UNIVERSITY

# Faculty Incivility

Carries implications for University processes and supports:

- "I would never confront a professor. I just wouldn't. I've said before though, I really dislike conflict. But I just wouldn't. They have so much influence over how you are doing in your schooling that I just wouldn't. A neutral party would be probably beneficial."
- "I talked to the president of the student union. [They] told me that [the Academic Member] had many complaints for being unfair to students, but nothing could happen because they are tenured. And then I had also heard they were good friends with the Dean so then I was like 'Oh great! I was extra hooped!' So now I'm just going to grit my teeth and bear it basically."

BRANDON UNIVERSITY

# Verbal Abuse

Bullying, sabotage, and rumors

Nurses:

- "…a lot of the people there were burned out … [and] didn't want students around, actively sabotaging, like that kind of stuff."
- "the nurse started this whole rumor that a student had pushed a patient…[as a result, the nurses] they pretty much stripped us down to being able to do *nothing*. Um, and just like lying about us."
- "…everybody acted like everything was sunshine. It was almost like a high school sort of situation where you know people are talking about you and saying bad things, but we all had to pretend that it's not happening.  It was *not* good. Very, very stressful two weeks."

BRANDON UNIVERSITY

# Verbal Abuse

Nurses:

- "We never knew if they were going to fly off the handle or just whatever, they didn't want to interact with us…. as students we can't ask the nurses, "Hey, are you burned out?" ah, so we could see it but we couldn't enact any change about it."
- "…there was kind of cattiness, which like, I understood, I knew what I was getting into and I knew I am going into a woman dominated profession…there is no like physicalness, but there is a lot of emotional things and nurses tend to eat their young."
- "…typically we would report with the nurse on our findings. But this one particular nurse she said, 'No, I'll do my report, and you do your report separate'… I felt like she just didn't see me as, umm, I don't know, somebody who was even there."

BRANDON UNIVERSITY

# Verbal Abuse

## Physicians:

- “He got this close to me [holds hand in front of face], and yelled at me in front of this family… At that moment I checked out, I wasn’t even in the room anymore. All I could think of was, I want to leave…like, what point did that make?  Was he trying to show he’s smarter than me…”
- “I just remember, the ICU doctor standing there screaming ‘Where’s the nurse, who knows this patient?’ And I remember having my little concept care map [a nursing care plan] and standing there… and I remember standing there, like, shaking.”
- “Doctors that yell at you, and sometimes that’s sometimes more traumatic that anything else, just being yelled at by somebody.”

# Verbal Abuse

From patients:

- “She was calling me constant names, some really, really, the worst things you could imagine, just constantly for an hour... I left the room and I was actually – like visibly shaking from the stress of being in the room.”
- [The patient] was, uh, really angry at me and screaming. And it was because they wanted some more lorazepam and when I asked them what was going on and they were, like, “Give me my Lorazepam!” [loud, yelling voice].
- “We had this behaviour patient who like stood at my med cart for the entire time I did meds and just screamed at me. She screamed in her room, then she’d run to the desk, and she’d scream at us at the desk for 12 straight hours.”

# Sexual Inappropriateness

From patients:

- "You get the 'grabby' patient…it's one of those things that it is nothing to write home about…I know its inappropriate, but nothing's going to come of it. They are drunk and grabby…One guy grabbed my butt, and working I've had guys grab my boob."
- "I would go to change his diaper because he was incontinent. And he would be like, 'Hey do you give hand jobs too?' I'm like, 'no [shakes head], no.' Or he'd be like, 'You got a nice ass on you!' And I got to the point where I just like, just keep walking, like I can't."
- "I'd tell my fiancé, 'yeah a guy today just tried to grab my ass'….it's part of my job. It shouldn't be, but it is. I had an old man try to punch me today and my fiancé is like, 'are you okay?' I'm like, 'yup.'" [stated with sarcasm].

BRANDON UNIVERSITY

# Exposure to Physical Violence

There were numerous accounts of patient-perpetrated or patient-affiliated violence disclosed:

- "…[the patient] took them from me and then threw the medications and the water cup at me. And I was like, 'Okay, I guess this is how we're starting the morning.'"

- "I called [my mom] after the shift, 'Oh yeah I had 5 patients today, one threw poop at me today, the others screamed at me today, I got another smoking meth in the bathroom today, I was sexually harassed all day by my brain injured patient.' She's like, 'Oh my God.'"

BRANDON UNIVERSITY

# Exposure to Physical Violence

Code White/Code Red [Aggressive & Violent Patient]:

- “We had to call a Code White and seclude him and give him a needle against his will. And that was the first time I saw that. That was pretty, like, um, it’s really intense the first time you see something like that happen, because it doesn’t feel humane at all…but it still affects you emotionally .”
- “…[a patient] lunged towards us… We ended up having to seclude him and giving him a needle, that kind of thing. But I was *so* afraid…. I don’t know, it was so scary.”
- “…she was scratching people, she was biting people, and then she got a pill crusher and was trying to hit people... So like, you call security and like you sedate them... you’re like traumatized after you’ve been abused by your patients.”

BRANDON UNIVERSITY

# Mobilizing Supports

Interpersonal Supports:

- "I'm thankful that my mom's a nurse. Because like after every shift, after every day shift I call her when I'm driving home…. I called my mom crying going, 'What have I done, I hate this floor, I hate my job, I've made a terrible mistake.'"
- "I did talk to her [my aunt] like a little while later about things… she just kind of heard me out and kind of validated some feelings I had without really actually saying a whole lot."
- "He [my partner] has no idea, like, he doesn't understand. He'll try and do his best but, um, I didn't want to put that on him because I chose to go into this profession."

# Mobilizing Supports

## Personal Health Behaviours:

- "I don't need sleep aids on my days off but when I'm working, it's like I actually need a sleep aid to make sure that I get my sleep so that I can get through the day."
- "…every week [I] would do one of the mindfulness practices… deep breathing and, uh, I should do more but its hard, I haven't done a body scan in a while, I do like colouring, um, I actually have a colouring book at my desk which is awesome."
- "I do things to cope in a day-to-day life. I go to the gym; I find that rewarding for myself. I'm very close with my family, I'm able to speak to them. I have a good friend group. I just live my everyday life."

# Mobilizing Supports

## University Institutional Supports:

- "It's really difficult to access help and services because, um, anytime we try to access any of those services, it's a fight because they don't realize that we even exist.... Even for somebody who was like on our student council, it was hard to navigate."
- "I've had a really bad experience with counselling and I think that really deterred me. And I was, like, "I'm okay. I'd rather not."
- "I'd try to go a couple of times but they were always full because it always happened to be around like exams and high stress times. So I tried to go in a couple of times and like even though they have like, there is like a few like critical walk-ins, I tried to go to those. And even those were full."

# Mobilizing Supports

## University Institutional Supports (cont'd):

- "I was feeling overwhelmed and lonely because I was living in a city where I didn't know anybody, didn't know anyone, and I had no friends here. And she [the counsellor] made me feel like other people have it worse off that you, you're fine."
- "I wanted to meet with one particular counsellor... but their availability wasn't for several weeks. [Tearing up]. I didn't feel like there was anybody I could talk to."

BRANDON UNIVERSITY

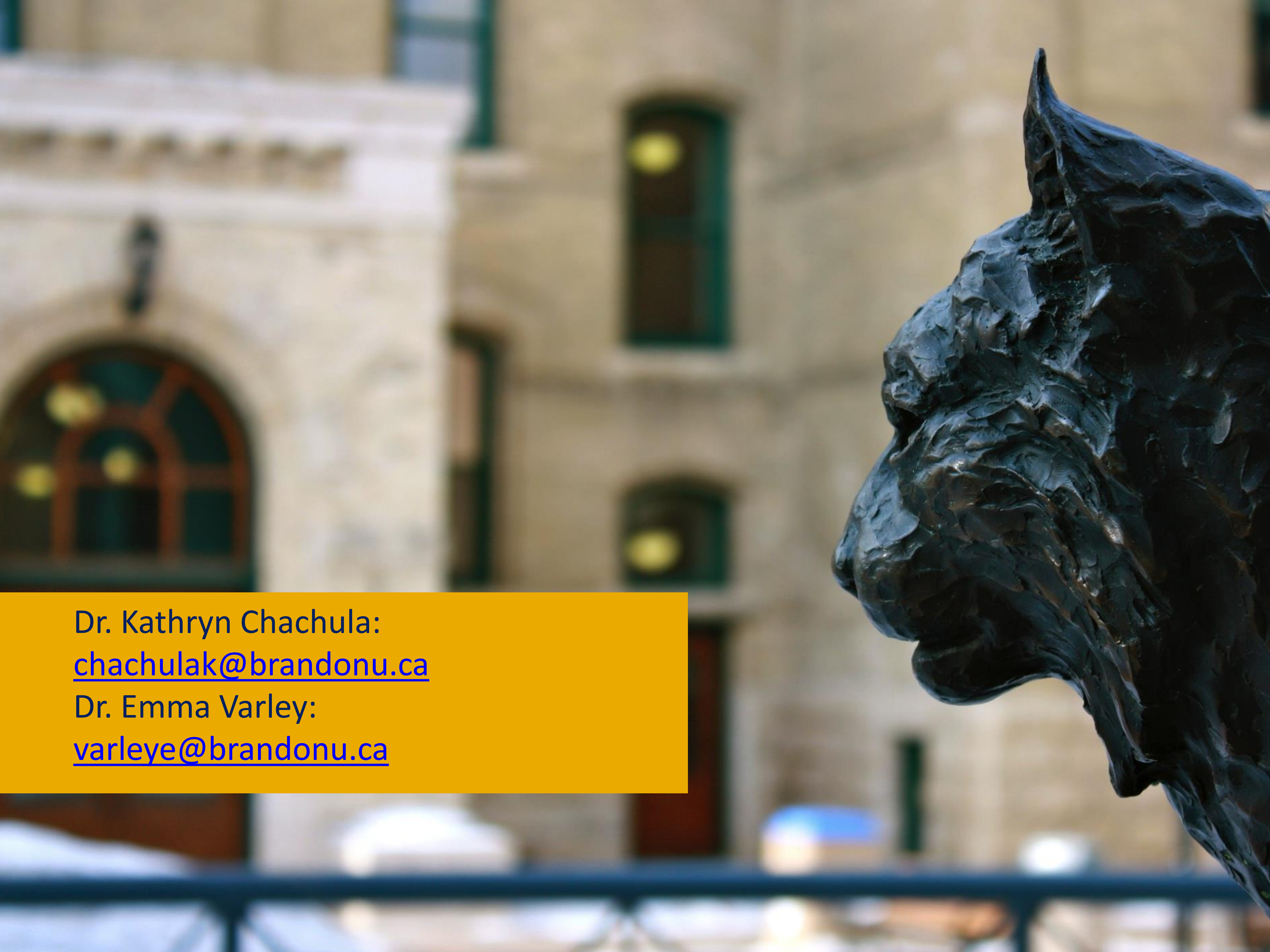

Dr. Kathryn Chachula:
chachulak@brandonu.ca
Dr. Emma Varley:
varleye@brandonu.ca
